# Blastomycosis Masquerading as Tuberculosis: A Diagnostic Challenge in a Patient With Diabetes

**DOI:** 10.7759/cureus.91774

**Published:** 2025-09-07

**Authors:** Gajendra Acharya, Rohit Pandit, Makondo Shimukowa

**Affiliations:** 1 Internal Medicine, TidalHealth Peninsula Regional, Salisbury, USA; 2 Infectious Disease, TidalHealth Peninsula Regional, Salisbury, USA

**Keywords:** blastomycosis, diabetes, diagnostic delay, disseminated fungal infection, endemic mycoses, ohio river valley, tuberculosis

## Abstract

Blastomycosis is an endemic fungal infection caused by *Blastomyces dermatitidis*. Immunosuppressed individuals, as well as those with diabetes mellitus or obesity, are more likely to develop severe disease. It is often referred to as “the great imitator” because it can closely mimic tuberculosis. Early diagnosis is critical to prevent delays in appropriate treatment and avoid complications. We present the case of a 51-year-old Haitian immigrant with poorly controlled diabetes mellitus who was initially diagnosed and treated for tuberculosis based on a positive interferon-gamma release assay (IGRA). Despite receiving RIPE (rifampin, isoniazid, pyrazinamide, and ethambutol) therapy, his symptoms failed to improve. Further comprehensive evaluation ultimately revealed disseminated blastomycosis with pulmonary and cutaneous involvement. He was successfully treated with liposomal amphotericin B, followed by itraconazole. This case underscores the diagnostic challenges of blastomycosis, particularly in immunocompromised individuals. In endemic areas, a high index of clinical suspicion and appropriate diagnostic testing are crucial to avoid misdiagnosis and to ensure timely and effective treatment.

## Introduction

Blastomycosis, caused by the dimorphic fungus *Blastomyces dermatitidis*, is a systemic fungal infection endemic to the Ohio and Mississippi River Valleys, the Great Lakes region, and parts of Canada. It is frequently misdiagnosed due to its wide range of non-specific symptoms, including fever, cough, and fatigue. Its ability to mimic other infections, particularly tuberculosis (TB), coccidioidomycosis, histoplasmosis, and leishmaniasis, complicates diagnosis, leading to delays in appropriate treatment [1,2].

The infection is often more severe in immunocompromised individuals, including those with poorly controlled diabetes mellitus, HIV/AIDS, and other conditions that impair immune response [3]. Early recognition of blastomycosis, particularly in its disseminated form, is essential to prevent morbidity and mortality. This case involves a Haitian immigrant with suspected TB and a positive interferon-gamma release assay (IGRA) who did not respond to RIPE (rifampin, isoniazid, pyrazinamide, and ethambutol) therapy and was ultimately diagnosed with disseminated blastomycosis, highlighting the challenges in differentiating between these two infections.

## Case presentation

A 51-year-old male patient, originally from Haiti, presented to the hospital with a month-long history of persistent symptoms, including hiccups, fever, chills, fatigue, vomiting, anorexia, and unintentional weight loss. The patient denied any history of chest pain, hemoptysis, or drenching night sweats. He immigrated to the United States in 2021, lived in Ohio for three years, and then relocated to Maryland. His medical history was significant for poorly controlled type 2 diabetes mellitus, evidenced by an HbA1c of 11.2% (<5.7% normal; 5.7%-6.4% prediabetes; ≥6.5% diabetes), associated with episodes of intermittent hyperglycemia and a prior hospitalization three months earlier for acute hypoxic respiratory failure. Given his immigration history from Haiti, a TB-endemic region, there was concern for an infectious etiology, including TB. His IGRA was positive, while sputum acid-fast bacilli (AFB) smears and cultures were negative, suggesting latent TB. He was started on the standard RIPE regimen. However, despite treatment, his symptoms failed to improve, and he continued to decline clinically.

**Figure 1:**
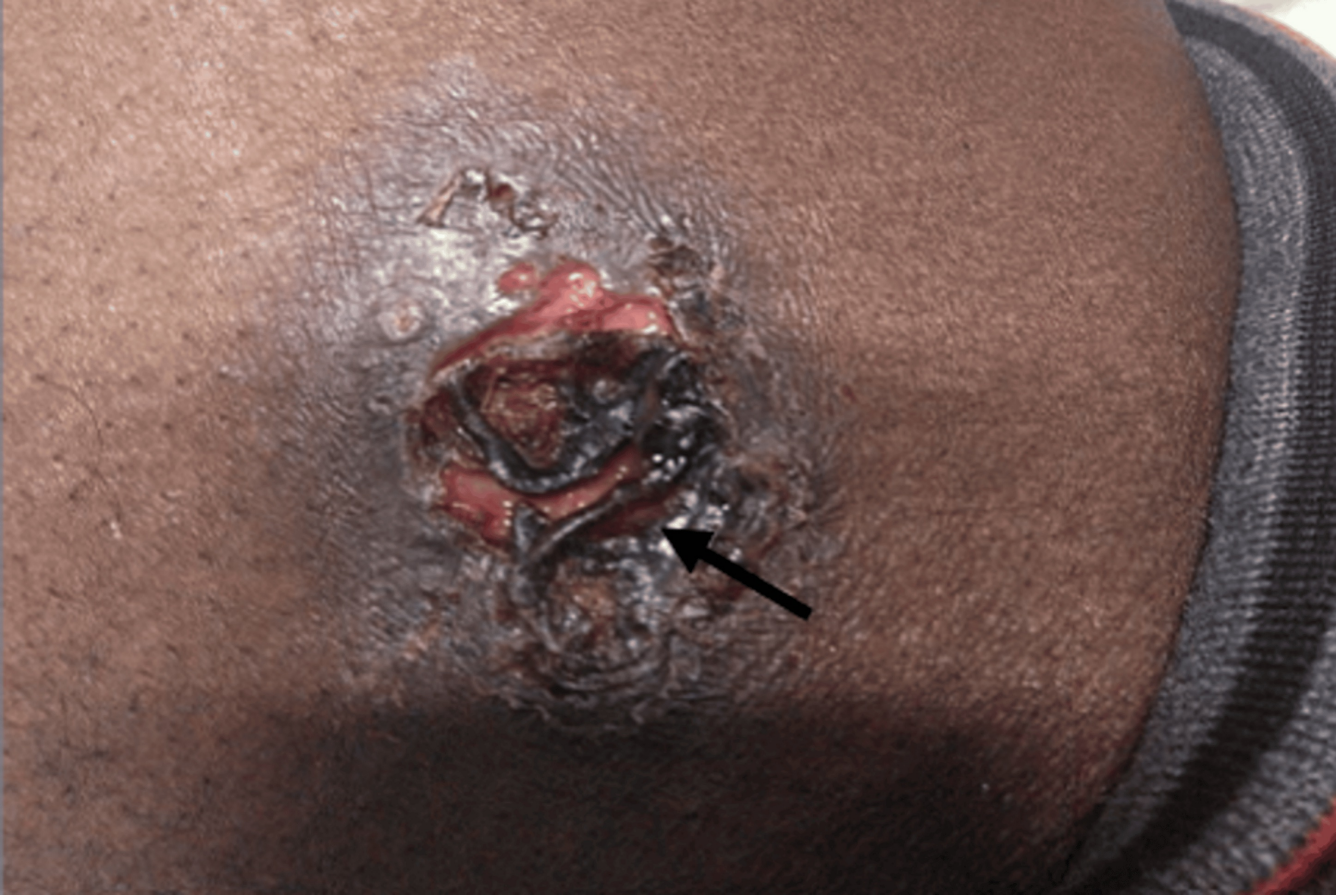
Necrotic ulcer on the lateral thigh characteristic of cutaneous blastomycosis. The lesion demonstrates a centrally necrotic eschar (arrow) surrounded by an irregular, raised erythematous border with surrounding induration. The ulcer base appears violaceous with satellite pustules. This presentation is consistent with disseminated blastomycosis, confirmed via fungal culture and histopathology showing broad-based budding yeast.

**Figure 2:**
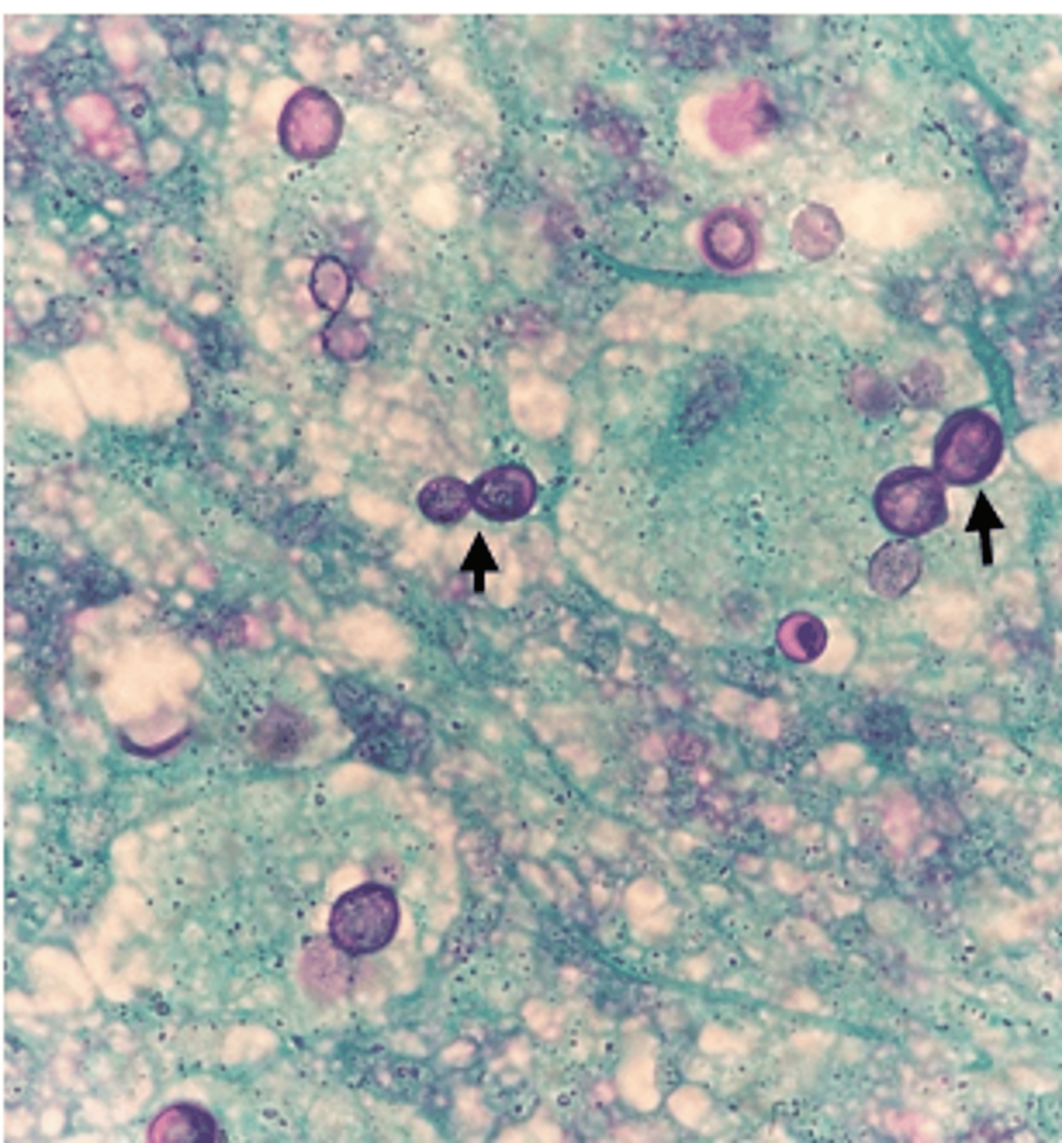
Periodic acid-Schiff stain (PAS) highlights broad budding yeast organisms (arrows). The histopathologic findings are consistent with blastomycosis.

**Figure 3:**
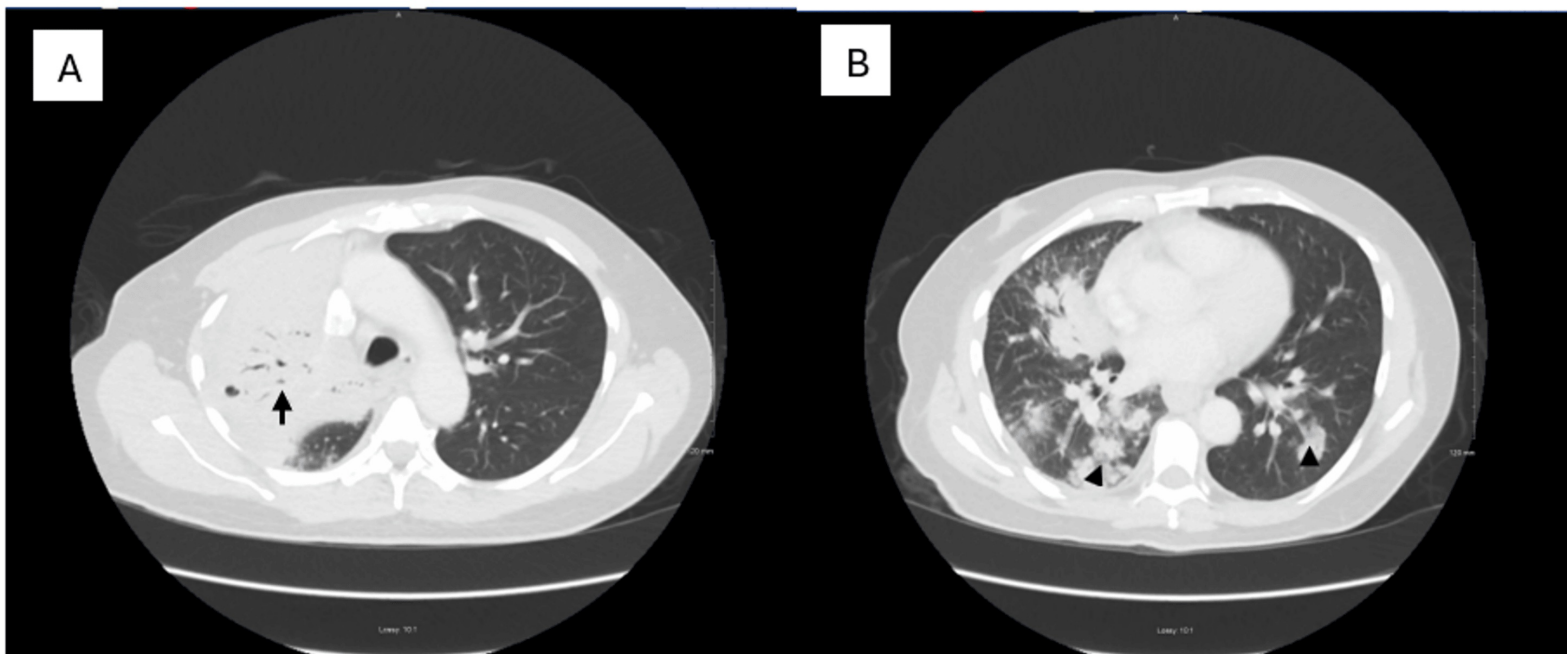
(A) Transverse chest CT image showing extensive consolidative changes in the right upper lobe (arrow) with mild mediastinal lymphadenopathy. (B) Patchy and nodular infiltrates are present in the bilateral lower lobes (arrowheads). These radiographic findings are consistent with pulmonary blastomycosis in the setting of systemic fungal infection.

**Figure 4:**
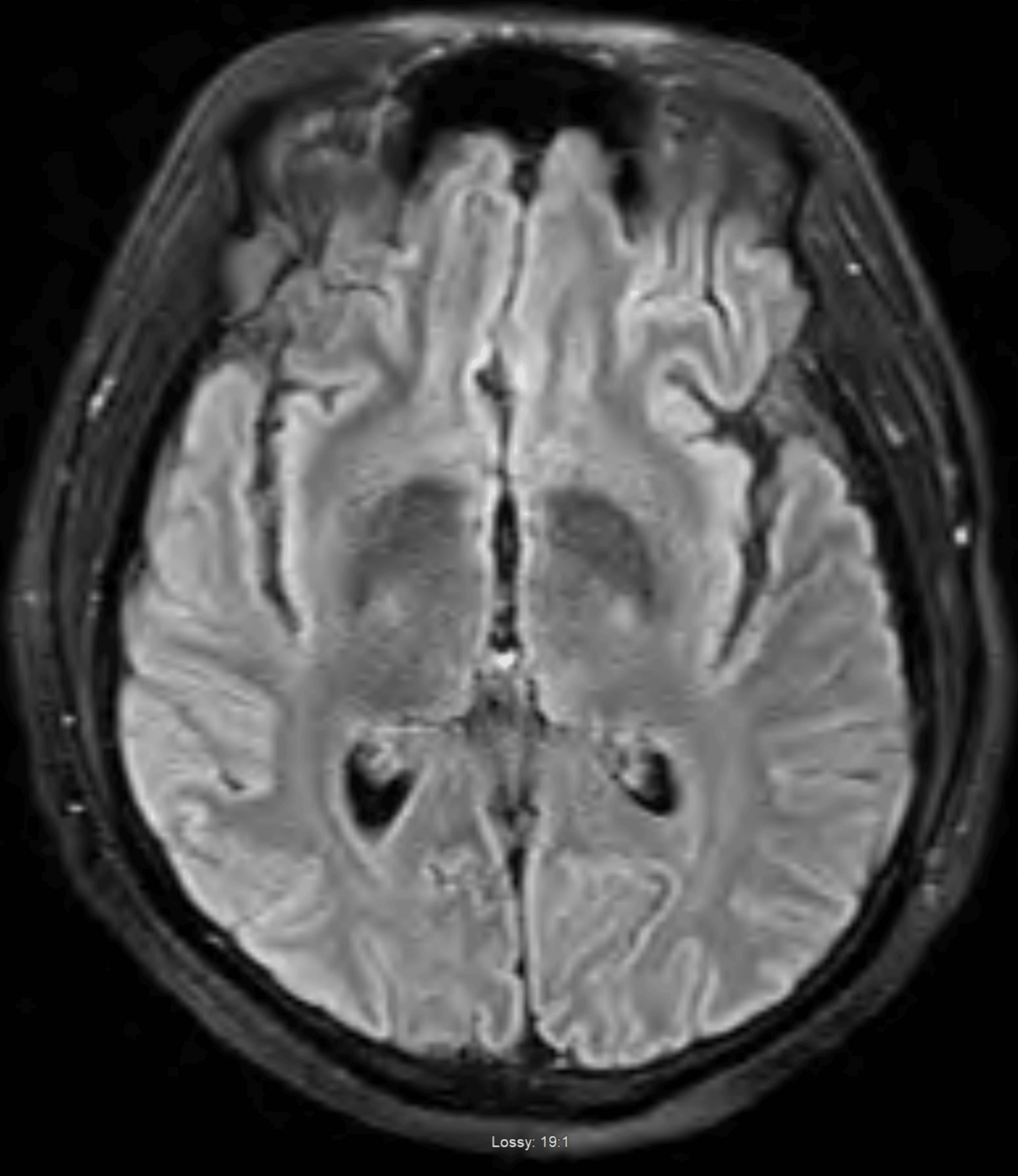
Axial MRI brain with and without contrast (T2 FLAIR sequence) showing no evidence of acute mass effect or cerebral edema. No space-occupying lesion is identified. FLAIR: fluid-attenuated inversion recovery

On further review of his clinical history, it was noted that he had emigrated from Haiti and settled in Ohio three years prior, an area recognized as endemic for *B. dermatitidis*. This prompted additional investigation, considering both the patient's geographical risk and clinical presentation. A new lesion appeared on his torso 3-4 weeks prior to presentation. It was slowly progressive, non-tender, non-painful, and without discharge, emerging after the onset of his initial symptoms. This lesion was subsequently biopsied (Figure 1). Pathological examination revealed broad-based budding yeast consistent with *B. dermatitidis*, pointing to a fungal rather than bacterial infection (Figure 2). Imaging studies were also conducted to assess pulmonary involvement. Chest radiography revealed bilateral infiltrates with consolidative changes, suggesting a disseminated infection (Figure 3). To rule out central nervous system involvement, an MRI of the brain was performed, which fortunately showed no evidence of intracranial pathology (Figure 4).

Given the identification of *B. dermatitidis* on biopsy, a diagnosis of disseminated blastomycosis was established. This was confirmed by the identification of the fungal organism in tissue samples and the clinical presentation, which included multi-system involvement with pulmonary and cutaneous manifestations. The decision was made to discontinue the RIPE regimen for TB and initiate treatment for blastomycosis. The patient was initiated on intravenous liposomal amphotericin B for its broad-spectrum activity against *Blastomyces* species, particularly in disseminated disease. After two weeks, treatment was discontinued due to thrombophlebitis, and he was transitioned to oral itraconazole with a planned 12-month course. This long-term antifungal therapy was selected to prevent the recurrence of disseminated blastomycosis.

**Figure 5:**
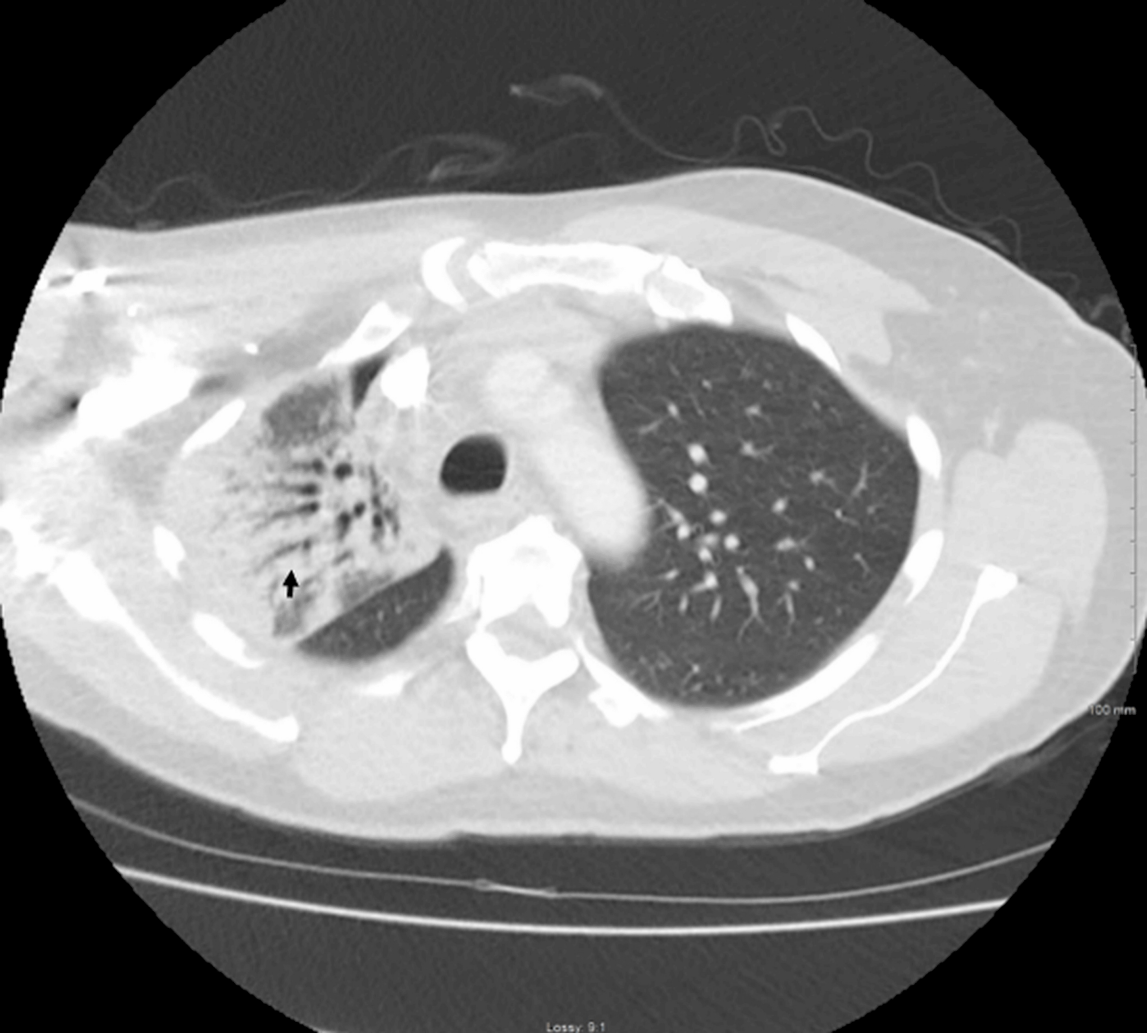
Transverse chest CT image demonstrating significant resolution of the right upper lobe infiltrative changes following treatment (arrow).

Over the course of treatment, the patient showed significant clinical improvement (Figure 5). His fever subsided, respiratory symptoms improved, and his cutaneous lesions gradually resolved. Regular follow-up visits were scheduled to monitor his progress, and liver function tests were closely monitored due to the potential hepatotoxicity associated with itraconazole. The patient remained compliant with the treatment regimen, and no further complications were reported during the follow-up period.

## Discussion

Blastomycosis presents a significant diagnostic challenge due to its clinical overlap with other pulmonary infections, especially TB. TB is endemic in many parts of the world, and patients from TB-endemic regions, such as the Caribbean, frequently present with symptoms that mimic fungal infections. In this case, the patient’s travel history and symptoms initially suggested TB, as IGRA is often positive in latent TB, which is common in endemic regions. However, persistent symptoms raised suspicion for fungal etiology.

Blastomycosis is particularly difficult to distinguish from TB due to their similar clinical presentations, including fever, cough, weight loss, and chest radiographic findings of pulmonary infiltrates or consolidation [1]. Imaging findings in blastomycosis often mimic those of bacterial pneumonia or TB, showing alveolar infiltrates, nodular opacities, or cavitary lesions [2]. The hallmark of blastomycosis, however, is its ability to disseminate, particularly in immunocompromised individuals, which was evident in our case with both pulmonary and cutaneous involvement.

Furthermore, as noted in the literature, the diagnostic delay in blastomycosis is common, with patients often being treated for other conditions such as TB or pneumonia before the correct diagnosis is made [4]. The delayed diagnosis in this patient led to an initial delay in the appropriate treatment, highlighting the need for clinicians in endemic areas to have a high index of suspicion for blastomycosis in patients with pulmonary symptoms who fail to respond to typical treatments. A distinctive feature of disseminated blastomycosis is the potential for cutaneous involvement, which can serve as an important diagnostic clue. Cutaneous lesions occur in approximately 30% of patients with disseminated blastomycosis and may be the first sign of infection [5]. The patient presented with a cutaneous lesion, for which the differential diagnosis included cutaneous TB, atypical mycobacterial infection, leprosy, leishmaniasis, and sarcoidosis. Histopathological examination of the biopsy specimen, however, confirmed the diagnosis of *B. dermatitidis* infection. This finding helped confirm the diagnosis of disseminated blastomycosis, which had initially been obscured by the clinical resemblance to TB. Cutaneous lesions in blastomycosis can range from ulcerative, verrucous lesions to more typical plaques or nodules. The identification of the characteristic broad-based budding yeast on histopathological examination is crucial for diagnosing blastomycosis [1]. It is essential for clinicians to recognize the potential for fungal involvement when cutaneous lesions are present, especially in endemic regions.

Immunocompromised individuals, such as those with poorly controlled diabetes mellitus, are at increased risk for disseminated blastomycosis. Diabetes mellitus, a condition characterized by impaired immune function, predisposes individuals to fungal infections by impairing neutrophil function, macrophage activity, and delayed-type hypersensitivity responses [6]. In our patient, poorly controlled diabetes likely contributed to the dissemination of the infection to both the lungs and skin, as well as to the development of secondary wound infections.

Although blastomycosis can affect healthy individuals, the risk of severe disease, including dissemination to organs such as the bones, joints, and central nervous system, is higher in those who are immunocompromised [1]. Diabetes mellitus has been associated with more severe outcomes in fungal infections, including blastomycosis, due to its effects on immune response [3].

The treatment of disseminated blastomycosis involves antifungal therapy, with liposomal amphotericin B being the drug of choice for severe or disseminated disease [7]. Itraconazole, an azole antifungal, is used for maintenance therapy after the initial treatment. In our patient, liposomal amphotericin B was administered initially, followed by itraconazole for 12 months.

Treatment with amphotericin B can be associated with nephrotoxicity and other side effects, including thrombophlebitis, which occurred in our patient. Switching to itraconazole after the acute phase of treatment allowed continued management with a more tolerable regimen. Monitoring liver function during itraconazole therapy is crucial, as hepatotoxicity can occur, especially in patients with comorbid conditions such as diabetes [7].

The patient showed significant improvement with treatment, including resolution of symptoms and healing of the cutaneous lesions. This outcome is consistent with the general prognosis for blastomycosis when treated promptly with appropriate antifungal therapy [8].

## Conclusions

Blastomycosis poses a significant diagnostic challenge, especially in immunocompromised patients, as it can closely mimic TB and other respiratory infections. Clinicians may anchor TB and overlook fungal etiologies, resulting in delayed diagnosis and potential complications. Early identification and antifungal therapy are critical to prevent morbidity and mortality, particularly in patients at high risk for disseminated disease, such as those with poorly controlled diabetes. This case illustrates the importance of considering endemic fungal infections in the differential diagnosis of TB-like presentations.

## References

[1] Saccente M, Woods GL (2010). Clinical and laboratory update on blastomycosis. Clin Microbiol Rev.

[2] Fang W, Washington L, Kumar N (2007). Imaging manifestations of blastomycosis: a pulmonary infection with potential dissemination. Radiographics.

[3] Smith JA, Kauffman CA (2010). Blastomycosis. Proc Am Thorac Soc.

[4] Alpern JD, Bahr NC, Vazquez-Benitez G, Boulware DR, Sellman JS, Sarosi GA (2016). Diagnostic delay and antibiotic overuse in acute pulmonary blastomycosis. Open Forum Infect Dis.

[5] Bradsher RW (1997). Clinical features of blastomycosis. Semin Respir Infect.

[6] Casqueiro J, Casqueiro J, Alves C (2012). Infections in patients with diabetes mellitus: a review of pathogenesis. Indian J Endocrinol Metab.

[7] Chapman SW, Dismukes WE, Proia LA, Bradsher RW, Pappas PG, Threlkeld MG, Kauffman CA (2008). Clinical practice guidelines for the management of blastomycosis: 2008 update by the Infectious Diseases Society of America. Clin Infect Dis.

[8] Khuu D, Shafir S, Bristow B, Sorvillo F (2014). Blastomycosis mortality rates, United States, 1990-2010. Emerg Infect Dis.

